# Using Delphi method to develop Chinese women’s cervical cancer screening intention scale based on planned behavior theory

**DOI:** 10.1186/s12905-022-02113-1

**Published:** 2022-12-10

**Authors:** Tingting Xin, Xian Ding, Han Gao, Chunting Li, Yuting Jiang, Xiao Chen

**Affiliations:** 1 School of Medicine, Jiangnan University, Wuxi, China; 2 Department of Anesthesiology, Affiliated Hospital of Jiangnan University, Wuxi, China

**Keywords:** Cervical cancer screening, Planned behavior theory, Scale, Screening intention

## Abstract

**Background:**

Cervical cancer is the most common malignant tumor in women with a high mortality rate. However, the awareness and participation of women in cervical cancer screening were not high, and rare attention was paid to cervical cancer screening. The extensive promotion and execution of cervical cancer screening in China are still facing difficulties. In order to fully comprehend and evaluate the barriers and promote factors of cervical cancer screening in women, the objective of this study was to develop a scientifically sound and clinically useful Chinese cervical cancer screening intention scale. This study would allow for the development of targeted interventions which may contribute to the increase of individual participation in cervical cancer screening going forward.

**Methods:**

This study used the Delphi method to construct a Chinese cervical cancer screening intention scale based on the theory of planned behavior (TPB) and evaluate its validity. The study was based on the overview of the TPB questionnaire proposed by Ajzen, and was conducted through the literature search and two rounds of the Delphi expert consultation. According to the literature search published from 2012 to 2022, the scale item pool was established and a questionnaire was designed. A survey of 16 experts from 6 different provinces, cities and regions in China was conducted, and the Delphi technique was used to collect and analyze expert opinions data.

**Results:**

The final scale consisted of 4 dimensions and 23 items. The response rates in two rounds of expert consultation were 80% and 93.75%, respectively, with authority coefficients of 0.928 and 0.930. Variation coefficients varied from 0.07 to 0.21. Dimensions included “attitude towards behavior”, “subjective norm”, “perceived behavioral control” and “behavioral intention”.

**Conclusions:**

Women’s cervical cancer screening intentions could be assessed with the scale, since it had high validity and reliability, as well as high authority and coordination, meanwhile affording explanations and improving the efficiency of interventions.

**Supplementary Information:**

The online version contains supplementary material available at 10.1186/s12905-022-02113-1.

## Introduction

Cervical cancer is still a serious public health concern, with 604,000 new cases and 342,000 deaths worldwide in 2020, 90 percent of which occurred in low- and middle-income countries [1]. With about 570,000 cases in 2018, cervical cancer was the fourth most frequent cancer in women, following breast cancer (2.1 million cases), colorectal cancer (0.8 million cases), and lung cancer (0.7 million cases) [2].

Cervical cancer posed a severe threat to women’s health in China, where there were over 60,000 annual fatalities and 110,000 new cases [1]. The human papilloma virus (hrHPV) is consistently thought to cause cervical cancer, based on substantial and consistent evidence, and high-grade precancerous lesions (e.g., cervical intraepithelial neoplasia [CIN] grades 2 and 3) can be the result of a persistent infection. Since, during the early stage, cervical cancer is asymptomatic, once diagnosed, it is mostly in the advanced stage, and advanced cervical cancer has a dismal prognosis and a high mortality rate. Fortunately, cervical cancer is one of the few cancers that has a known cause and is curable when caught early. As cervical cancer is a preventable and controllable malignant tumor, cervical cancer screening can detect abnormal cells and allow treatment before they become cancerous [3]. Research suggested that effective cervical cancer screening and treatment can cut the disease’s incidence and mortality by 60% to 90% [4]. Population based screening programs, as well as HPV vaccination programs, contributed to the decrease of the incidence of cervical cancer. The survey [5] showed a 63% reduction in invasive cervical cancer among women who received the quadrivalent HPV vaccine, and an even greater 88% reduction among women who received the vaccine before age 17. However, compared with high income countries, low income countries had a 35% higher risk of cervical cancer due to factors such as a shortage of screening facilities, financial concerns, cultural factors, and insufficient awareness that limited the availability of cervical cancer screening [6, 7]. Reportes indicated [8, 9] that in developing countries like China, the screening rate of cervical cancer was generally low due to various factors. Most women have a herd mentality, and the people around them are less screened, so they think they have no problems and do not need to be screened. Rural women have a higher willingness to screen but a lower willingness to pay. The high cost of screening was one of the reasons for the decrease of patients’ will to participate in cervical cancer screening [9]. The low cost of screening can be accepted by more women, and the frequency of screening can be increased. The availability of health insurance was also a major factor influencing the utilization of screening services, and women with health insurance had higher utilization of screening services [10]. The survey found that [11] women with free medical care were more active in participation in cervical cancer screening than women who paid for it at their own expense, but the difference in actual screening behavior was not significant. Although the progress of free cervical cancer screening was significant, the participation rate still needed to be improved due to the limited funds and manpower and material resources of free screening. Therefore, it is particularly urgent to identify the behavioral mechanism and impact mechanism of cervical cancer screening.

The theory of planned behavior (TPB) [12], an extension of the theory of rational behavior, is a cognitive-motivated behavior theory with five main components: attitudes toward behavior, subjective norms (the influence of significant others on individual behavioral decisions), perceived behavioral control, the behavioral intention to perform the behavior, and the behavior actually performed. The core point of this theory is that people’s intention to engage in behavior is a key factor in determining whether such behavior occurs, and attitudes, subjective norms, and perceived behavioral control over behavior can predict behavior an individual will engage in or perform. The theory was first put forward by Ajzen, and it is a theory to predict and understand specific behaviors in a specific context.

The theory of planned behavior (TPB) can explain its determinants, and inform strategies to increase its adoption/compliance. TPB was applied to screening behaviors, such as breast cancer screening, colorectal cancer screening, and prostate cancer screening behavior [13]. It has also been found to be effective in predicting the willingness of cervical cancer screening in previous studies [14, 15]. Women’s decision-making is the result of internal factors, and the decision-making process reflects people’s values and attitudes. Therefore, understanding women’s motivations for screening can help strengthen screening and treatment efforts.

At present, the research on cervical cancer screening behavior tends to use behavior change theory to investigate the cognitive influences on women’s cervical cancer screening, and to strengthen the individual’s intention and behavior to participate in cervical cancer, which is conducive to the adoption of targeted health management. However, the current research on the related factors of cervical cancer screening is limited to simple knowledge, or the behavioral research on cervical cancer screening mostly focuses on the knowledge, attitude and practice model [16, 17] and the health belief model [18]. The low number of actual cervical cancer screening behaviors can be explained rationally using the notion of planned behavior.

An intention scale for cervical cancer screening participation was developed in this study using the Delphi method in the Chinese population. According to the literature and expert opinions, this manuscript aimed to better understand and predict women’s intention to undergo cervical cancer screening and sense of control, and to help prevent and detect the disease, thereby providing information and decision-making basis for carrying targeted preventive interventions in the future.

## Methods

### Design

#### Initial construction of TPB scale item pool for cervical cancer screening

**Fig. 1:**
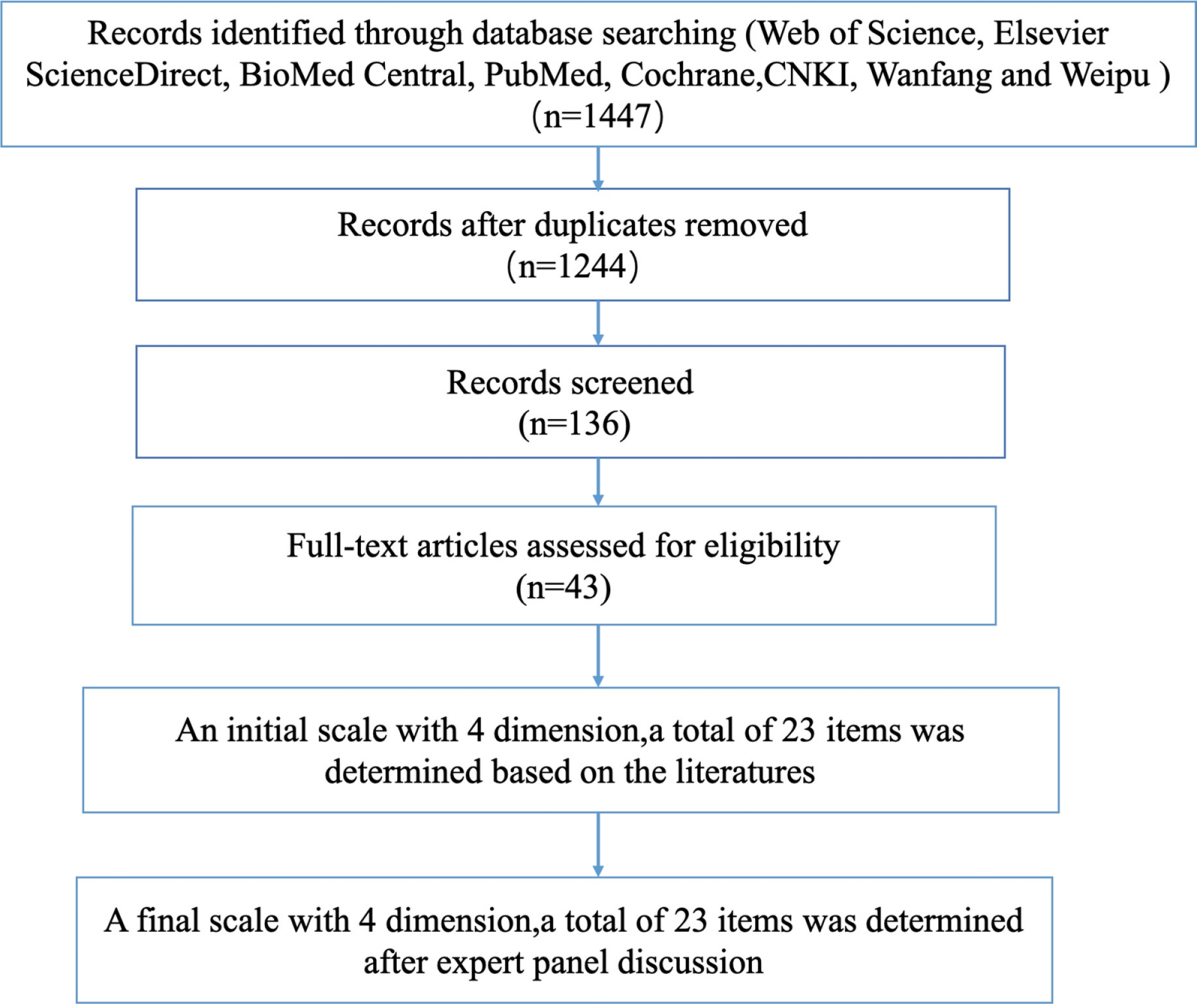
Flow chart of literature search and initial item identification of TPB scale for cervical cancer screening

The development of the scale first followed the process outlined by Ajzen, creating four behavioral predictors (attitude towards behaviors, subjective norms, perceived behavioral control, and behavioral intentions), A literature search was then performed to construct a project pool in the predictors, using international databases and Chinese databases including Web of Science, Elsevier ScienceDirect, BioMed Central, PubMed, Cochrane, CNKI, Wanfang and Weipu. The following search terms were used to find articles published in the past decade from 2012 to 2022: cervical cancer screening, participation, influencing factors, intentions, motivations, attitudes. English-and Chinese-language articles could be included. A total of 1447 original papers were obtained. After removing duplicates, 136 papers were screened and 43 papers were chosen to create the evaluation scale. It was determined according to the article title, abstract and content. Each paper had two screenings. In case of disagreement, members of the research team would discuss and decide together. References were managed using the Zotero software. Taking into account the references and discussions in the research group, the research team developed a pool of potential projects based on China’s current situation. Finally, the initial TPB intention scale for cervical cancer screening was constructed with 23 items including 4 dimensions. The flow chart shown in Fig. 1 illustrated the steps taken to search literature and identify items of the TPB intention scale for cervical cancer screening.

### Delphi survey

Using a series of questionnaires and evaluations by experts, the Delphi method is a structured method for achieving consensus on a topic. Using this approach, we communicated two rounds of questionnaires to get feedback from experts and corresponded with them via email and WeChat. The Delphi questionnaires were administered as online questionnaires (e-Delphi). On the basis of expert evaluation and discussion, a TPB intention scale for cervical cancer screening was established. The original Delphi method was modified to allow experts to add/modify items.

The research team consisted of 5 members, including 2 Gynecological clinician experts, 2 nursing associate professors, and 1 nurse in charge, who provided expertise and participated in decision-making and discussions in the development of the questionnaire.

There were three sections in the questionnaire: an introduction, the main body, and details on the experts. (1) The goal and history of this study were mostly discussed in the introduction. Additionally, it had thorough instructions on how to complete the questionnaire. (2) The second section was the body and contained the scale’s evaluation information. It assessed the project’s significance using a Likert five-point scale. According to the five-point Likert scale, 5 was very important, 4 was important, 3 was average, 2 was not very important, and 1 was least important. The expert can express his or her opinion on the item, add, amend, or eliminate the risk factor and explain the pertinent reasons. Items with a score of 4–5 indicated that the expert supported the item. The following criteria must be met for an item to be included: The item was endorsed by 70% of the experts; mean item importance score > 3.5; coefficient of variation < 0.30. (3) Experts’names, positions, and titles were collected among their personal information.

Two rounds of surveys were used to perform the Delphi study. According to a review of the literature, the initial round of questionnaires was created. The study team added and removed some things from the scale based on professional opinions after the initial round of enquiries via email or the social media platform WeChat. Feedback information was provided to the expert members through email and WeChat communication for the second round of survey, and the final cervical cancer screening TPB scale was formed, with a total of 23 items.

### Participants

There were 16 experts from six different provinces and regions. The criteria for experts participating in this study were as follows: (1) relevant working years in the field of gynecology clinical or education ≥ 10 years (2) intermediate title or above; (3) bachelor degree or above; (4) interested in this research, informed consent and those who can complete two rounds of correspondence. (5) Experts specialized in clinical medicine (gynecology), nursing, or public health.

### Data analysis

Data processing and analysis were performed using SPSS26.0 statistical software. The coefficient of variation was reported with the descriptive analysis. The degree of expert opinion dispersion was determined using the coefficient of variation. Frequency and percentage (%) were used to express the count data. The questionnaire recovery rate was used to gauge experts’ enthusiasm, while the expert authority coefficient (Cr) was used to gauge their level of authority. The coefficient of variation (CV) and the Kendall coordination coefficient (W) measured the degree of expert opinion coordination. The lower the coefficient of variation (CV), the better the expert opinion coordination. W denoted the degree to which all experts coordinate across all indicators, and its value ranged from 0 to 1. A statistically significant difference is indicated by *P* < 0.05 [19].

### Ethical consideration

The Jiangnan University School of Medicine Ethics Committee approved this study. All participants provided verbal informed consent.

### Validity and reliability

The expert group was made up of university professors, nurses, and clinical gynecology experts from developed and developing cities across the country, from 9 hospitals and 2 universities spread across 6 cities. Delphi research was conducted to demonstrate the validity and reliability of the research. Experts had the authority to add, change, or remove risk factors. Two rounds of investigation were carried out until all expert opinions agreed.

## Results

### Demographic description of experts

The study included 16 experts in total. In terms of age, 37.5% of experts were younger than 40 years old, 40–50 years old accounted for 37.5%, and 4 experts were over 50 years old, accounting for 25%. In terms of educational background, there were 6 bachelor’s degrees (37.5%), 3 master’s degrees (18.75%), and 7 doctor’s degrees (43.75%). In terms of professional fields, the experts were occupied with clinical medicine (62.5%), and nursing (31.25%), or public health (6.25%). The specific demographic information for specialists was shown in Table 1.

**Table 1:** Demographic characteristics of experts (n = 16)

Characteristics	*N*	Percentage (%)
Gender		
Male	5	31.25
Female	11	68.75
Age(years)		
< 40	6	37.5
40–50	6	37.5
> 50	4	25
Level of education		
Bachelor	6	37.5
Master	3	18.75
PhD	7	43.75
Title		
Intermediate title	2	12.5
Associate Professor	10	62.5
Professor	4	25
Professional field		
Clinical medicine	10	62.5
Nursing	5	31.25
Public Health	1	6.25
Years of work experience		
10–15	4	25
16–20	5	31.25
> 20	7	43.75

### Round 1

The response rate of this round of experts was 80%. According to the results and suggestions of the first round of correspondence, it was revised to 4 first-level indicators and 23 s-level indicators. In the first round of the survey, firstly, the expert suggested deleting “I think regular cervical cancer screening is beneficial for obtaining health” and “I think it is better to detect cervical cancer earlier than later” in “Attitude towards behavior”. Secondly, the expert believed that the “husband” and “family” in “subjective attitude” were repeated. Next, some experts proposed that “I can accept the diagnosis result of cervical cancer screening” in “perceived behavioral control” should be amended. What’s more, one expert suggested “I intend to undergo cervical cancer screening in 3 months” and “I will definitely undergo cervical cancer screening in the next 3 months” in the “behavioral intention” should be merged and modified. In addition, some items were suggested to be added, including “I will regret if I miss or do not participate in the cervical cancer screening program” in “Attitude towards behavior”, “I am willing to listen to suggestions arranged by the community/company to do cervical cancer screening” in “Subjective norm”, “I will definitely have regular cervical cancer screening” in “Behavioral Intentions”. Statements removed and added after round 1 were shown in Table 2.

**Table 2:** The statements that were changed after round1—those that were removed, added, or combined

After round 1, four deleted statements	
Attitude towards behavior	(1) I think regular cervical cancer screening is beneficial for health
	(2) I think it’s better to find cervical cancer sooner rather than later
Behavioral intention	(3) I plan to have a cervical cancer screening in three months
	(4) I will definitely have a cervical cancer screening in the next three months
After round 1, one added statement	
Attitude towards behavior	(1) I will regret if I miss or do not participate in the cervical cancer screening program
Subjective norms	(2) I am willing to listen to suggestions arranged by the community/company to do cervical cancer screening
Behavioral intention	(3) I would like to undergo cervical cancer screening if I can in the future
	(4) I will definitely have regular cervical cancer screenings

### Round 2

The response rate of this round of experts was 93.75%. The experts did not mention any additions or deletions in the second round of expert consultation. Items with a CV value greater than 0.25 points or an average value less than 3.5 points were removed. The research team revised the project and eventually developed a preliminary cervical cancer screening intention TPB scale with four first-level items and twenty-three second-level items. The responses were divided into five levels of intensity, including strongly disagree, disagree, uncertain, agree, strongly agree. The final scale was shown in Table 3.

**Table 3:** The final planned behavior theory cervical cancer screening intention scale

Dimension 1: Attitude towards behavior	
1. I think it is important to get information about cervical cancer screening 2. I feel that having regular cervical cancer screenings gives me peace of mind about my health 3. I think cervical cancer screening can help decrease the incidence 4. I think cervical cancer screening can help reduce the death rate of women 5. I think cervical diseases can be detected early through cervical cancer screening 6. I will regret if I miss or do not attend a cervical cancer screening program 7. I believe that all eligible women should be screened regularly for cervical cancer Dimension two: subjective norms 1. My boyfriend/husband will support me if I get screened for cervical cancer 2. My family thinks I should be screened for cervical cancer 3. My friends encourage me to get screened for cervical cancer 4. I am willing to listen to the advice of people around me to get cervical cancer screening 5. For me, the doctor's advice is very important in my cervical cancer screening 6. I am willing to listen to the suggestions of the community/unit to do cervical cancer screening Dimension three: perceived behavioral control 1. I think it is entirely up to me to decide whether to get screened for cervical cancer 2. Doing cervical cancer screening will make me feel embarrassed/ashamed 3. I'd be more willing to be examined by a female doctor 4. I will be screened for cervical cancer even if I am healthy 5. I will be screened for cervical cancer despite some discomfort during the screening process 6. If cervical cancer screening becomes more convenient, I will get screened 7. If cancer is found, I choose not to know Dimension four: behavioral intention 1. After knowing the relevant information, I am willing to undergo cervical cancer screening 2. I would like to undergo cervical cancer screening if I have the conditions in the future 3. I will be screened regularly for cervical cancer	

### Authority and coordination

The answers to the questionnaire had a major role in determining the authority of experts. A Delphi survey and two rounds of expert questionnaires were undertaken for this investigation. In the first round, 20 questionnaires were distributed, with 16 recovered, for an effective recovery rate of 80%. In the second round, 16 questionnaires were distributed, with 15 recovered, for an effective recovery rate of 93.75%. Expert authority coefficient (Cr) = (judgment basis (Ca) + expert self-evaluation (Cs)) /2. The authoritative coefficients Cr of the two rounds of surveys were 0.928 and 0.930, indicating that the experts involved in this study held positions of great authority. The coefficient of variation (CV) and Kendall coordination coefficient (W), which together showed the agreement of expert opinions. The coefficient of variation (CV) varied from 0.07 to 0.21. Kendall coordination degree (W) were 0.198 and 0.247, respectively. This demonstrated a high consensus among all experts on the findings. Table 4 displayed the degree of survey coordination between the two rounds.

**Table 4:** Coordination of expert opinions in two rounds of surveys

Round	Kendall degree of coordination (W)	χ^2^	*P*
1	0.198	69.667	< 0.01
2	0.247	81.673	< 0.01

## Discussion

The Delphi technique was utilized in this study to design a scale based on planned behavior theory that can be used to measure Chinese women's intentions regarding cervical cancer screening.

In this study, the TPB intention scale for cervical screening demonstrated high reliability. The two rounds of surveys’ authoritative coefficients (Cr) were 0.928 and 0.93, respectively, showing that the experts participated in this research had a lot of authority. It is generally acknowledged that reliability is better when the authority coefficient Cr is higher than 0.70. Kendall’s coefficient of agreement W for the two rounds of the survey was 0.198 and 0.247, respectively, showing that there was strong consensus among all experts.

The use of the Delphi method, involving experts from multiple regions, increased the effectiveness and universality of the content. Conducting online surveys with experts through email contact and WeChat individual communication, prevented interaction between participants and domination and mutual influence by individual group members.

The Theory of Planned Behavior is the most widely used model for predicting intention [19], and a precursor to behavior, with widespread use [20, 20]. In this study, the scale was designed from the aspects of attitude towards behavior, subjective norm, perceived behavioral control, and behavioral intention, so that the investigation and intervention of cervical cancer screening intention in the future could have rules to follow.

“Attitude towards behavior” refers to the positive or negative evaluation of cervical cancer screening, attitude is also a significant predictor of intention, and “Subjective norm” refers to the level of social pressure felt about whether or not to get screened for cervical cancer, that is, to support significant others who want to be screened for cervical cancer, and if their significant other agrees or encourages screening, they more likely to intend to participate in screening. “Perceived behavioral control” refers to the degree of ease or difficulty of cervical cancer screening perceived by the screening population, which reflects the screening population’s cognition of promoting or hindering cervical cancer screening behavior. If women feel that they can perform screening well, they are more likely to participate in screening. This theory states that an individual’s intention to engage in or carry out an action can be predicted by attitude toward behavior, subjective norms, and perceived behavioral control. In turn, intention directly predicts whether an action will be carried out.

Despite there were some advantages to using the Delphi method to gain expert opinions and consensus, our study existed some limitations. First of all, the selected items in subjective norms section largely covered injunctive norms (how significant others would judge me if I participated in the screening program), but did not cover descriptive norms (did significant others participate in screening?). What’s more, during the compilation of this research scale, only expert data from six regions of the country were collected, which had certain regional limitations. It’s possible that the findings didn’t accurately reflect all Chinese people. Whether the conclusions are applicable to national promotion remains to be confirmed by further research. In the future, it is necessary to conduct external verification of the newly developed scale and conduct multi-center survey with sufficient sample size to verify this study.

## Conclusions

In summary, this research successfully developed a TPB intention scale for cervical cancer screening in China by the Delphi method. The scale consisted of 4 dimensions and 23 items. The scale’s significant validity and reliability were ensured through two rounds of Delphi expert consultation, which had a high level of authority and coordination. It offered instructions for forecasting women’s intentions to screen for cervical cancer and starting focused interventions in the future (Additional file 1).

## Supplementary Information

**Additional file 1:** The 43 papers chosen as a source.

## Data Availability

The authors confirm that all the data supporting the findings of this study are included in this published article.

## Abbreviation

TPB: The theory of planned behavior
