# Supplementary material for: Using Delphi method to develop Chinese women’s cervical cancer screening intention scale based on planned behavior theory
Source: BMC Womens Health. 2022 Dec 10;22:512. doi: 10.1186/s12905-022-02113-1 (PMC9741791; doi:10.1186/s12905-022-02113-1)
Supplement: Supplementary file 1 — Additional file 1: The 43 papers chosen as a source. [file 12905_2022_2113_MOESM1_ESM.docx]

1. Dsouza JP, Broucke SV den, Pattanshetty S, Dhoore W. A comparison of behavioural models explaining cervical cancer screening uptake. BMC Womens Health. 2022 Jun 16;22:235.

2. Enyan NIE, Davies AE, Opoku-Danso R, Annor F, Obiri-Yeboah D. Correlates of cervical cancer screening participation, intention and self-efficacy among Muslim women in southern Ghana. BMC Womens Health. 2022 Jun 13;22(1):225.

3. Hu Z, Sun Y, Ma Y, Chen K, Lv L, Wang L, et al. Examining Primary Care Physicians’ Intention to Perform Cervical Cancer Screening Services Using a Theory of Planned Behavior: A Structural Equation Modeling Approach. Front Public Health. 2022;10:893673.

4. Wollancho W, Amdissa D, Bamboro S, Wasihun Y, Tareke KG, Gizaw AT. Determining behavioral intention and its predictors towards cervical cancer screening among women in Gomma district, Jimma, Ethiopia: Application of the theory of planned behavior. PLoS One. 2020;15(11):e0238472.

5. Zhang J, Sha Z, Gu Y, Li Y, Yang Q, Zhu Y, et al. Predicting Behavioral Intentions Related to Cervical Cancer Screening Using a Three-Level Model for the TPB and SCT in Nanjing, China. Int J Environ Res Public Health. 2019 Oct;16(19):3575.

6. Yang W, Tian T, Wan D, Xu L, Wu Q, Chen J, et al. Survey on status of knowledge, attitude, and practice about cervical cancer prevention and control and its influencing factors among Chinese women. Health Education in China. 2017;33(10):867-880（in Chinese）.

7. Shao Y, Yang Y, Qin M, Shi Q, Chen Y. Breast and Cervical Cancer Screening Rates and Influencing Factors Among Adult Women in Yunnan Province. China Tumor. 2019;28(11):821-826（in Chinese）.

8. Liu J, Zhang yingying. Status quo of screening and KAP of cervical cancer for women in rural areas of eastern Henan province. Nursing Research. 2019;33(05):839-842（in Chinese）.

9. Cao L, Yang L, Cheng X, Zhu P. Analysis of anxiety status and its influencing factors of women of childbearing age before cervical cancer screening. Nursing Practice and Research. 2021;18(24):3645-3649（in Chinese）.

10. Wang H, Xu Z, Zhao P. Status and analysis of knowledge, attitude and practice related to cervical cancer screening among married women in Yantai. Modern Preventive Medicine. 2019;46(06):1031-1034，1058（in Chinese）.

11. Lu J, Huang Y, Zhu M. The analysis of knowledge level and related influencing factors of cervical cancer screening among women of childbearing age in Shihezi, Xinjiang. Nursing Practice and Research. 2022;19(10):1436-1441（in Chinese）.

12. Pan X, Zhang M, Huang X, Yang J. Multiple factors correlation analysis on influencing factors of cervical cancer screening for women in Xiqiao town. China Medical Sciences. 2020;10(14):176-180（in Chinese）.

13. Wang Y, Zhang Y, Peng J. Survey of Knowledge Awareness and Willingness to Cervical Cancer Prevention and Control in Female Screening Participants in Shenzhen. China Tumor. 2015;24(12):981-984（in Chinese）.

14. Wang L, Tao P. Cognitive survey of cervical cancer and related knowledge among community women. Shanghai Pharma. 2020;41(06):17-20,24（in Chinese）.

15. Yu J, Zhang H, Wu Z, Li M. Analysis of willingness to participate in cervical cancer screening among floating women in Xiamen and its influencing factors. Journal of Hainan University (Medical Science). 2022;41(01):68-72(in Chinese).

16. Chen X, Wang X, Luo X, Liu J, Qi C, Zhang R. Investigation of cognitive behavior of cervical cancer screening in pregnant women. China’s Family Planning and Obstetrics and Gynecology Department. 2018;10(02):67-71(in Chinese).

17. Zhang X, Ma G. Analysis of the factors related to the cognition, attitude and behavior of rural women with positive cervical cancer screening. Maternal and child Health in China. 2015;30(32):5640-5642(in Chinese).

18. Xia J, Luo X, Xue S, Mao L, Chen T. The obstacles of women in rural area taking part in screening of cervical cancer and stragies. Chinese Journal of Maternal and Child Health. 2012;3(05):263-266(in Chinese).

19. Huang J, Yang X, Liu A, Zhou W. Problems and Countermeasures in the Implementation of National Cervical and Breast Screening Program for Women in Rural Areas. General Practice in China. 2020;23(13):1680-1686(in Chinese).

20. Zhang Y, Wu X, Sun G, Wu X. Investigation on incidence and related factors of cervical cancer among women in farming and pastoral areas of a county. Chinese Medical Guide. 2017;15(13):81-82(in Chinese).

21. Ma G, Wang Y, Yan Y, Wang X. Investigation on cognition of cervical cancer，HPV-related knowledge and attitude among rural middle-aged women in Wudu District, Longnan City. Health Education in China. 2016;32(09):818-821,829(in Chinese).

22. Mu H, Yu L, Li Y, Liu L, Zhan X, Meng F, et al. Analysis of breast cancer and cervical cancer screening and influencing factors among urban and rural female residents in Liaoning province. Public health Management in China. 2015;31(02):197-198,201(in Chinese).

23. Zhao J, Liu Y, Li Q, Huo Z, Yang L, Duan L. Investigation and analysis of the cognition, attitude and behavior of female teachers on cervical cancer screening in Kaiyuan city. Maternal and Child Health in China. 2014;29(18):2960-2961(in Chinese).

24. Shen J. Opportunistic screening for cervical precancerous lesions in postmenopausal women and its influencing factors. Maternal and Child Health in China. 2022;37(08):1482-1485(in Chinese).

25. Meng X, Zhang H, Yang N, Li Z. Analysis of knowledge and service utilization behaviors related to cervical cancer screening among migrant female workers creening among migrant female workers. Health Education in China. 2016;32(10):915-918(in Chinese).

26. Chen Z, Zhang Q, Wang Q. Coverage and associated factors of cervical and breast cancer screening among childbearing women in Jilin province. Public Health in China. 2017;33(08):1170-1173(in Chinese).

27. Sun D, Zhang J, Zhang X, Li C, Jiang R, Ban L, et al. Research on the influencing factors of women’s cervical cancer screening behavior in Jinan based on the health belief model. Modern Preventive Medicine. 2021;48(19):3600-3604(in Chinese).

28. Luo Y, Yuan Y, Zhou Y, Fu J, Chen M, Wang T. Analysis on the status and influencing factors of breast cancer and cervical cancer knowledge among the women in the poor areas of Hu’nan Province. Maternal and Child Health in China. 2020;35(17):3130-3134(in Chinese).

29. Li Q, Liu Q, Bai Y, Yu Q, Zhang M, Gao Y, et al. Analysis on cervical cancer knowledge and screening attitude in Han and Tujia women in Wufeng rural area of Hubei Province. Health Education in China. 2019;35(08):722-726(in Chinese).

30. Fu M, Wang L. Investigation on cognition and willingness for screening cervical cancer among female patients in Banshan town of Hangzhou. Public Health Management in China. 2018;34(03):368-370,373(in Chinese).

31. Luo Q, Luo Y, Gong Y, Lao H, Cao X, Huang C, et al. Cognition and Screening Behaviors and Related Influencing Factors of Cervical Cancer among Rural Women in Hainan Province. Chinese Journal of Social Medicine. 2020;37(01):74-78(in Chinese).

32. Wu T, Han B, Fu J, Wang B. Investigation the situation of knowledge-attitude-practice on cervical cancer screening of rural women in Haikou. Chinese Journal of Family Planning. 2020;28(12):1950-1954(in Chinese).

33. Liang W, Guo L, Li M, Zheng Z, Wu C. Analysis of the factors affecting cervical cancer screening in the community population of Guangzhou City via a structural equation model. Chinese Journal of Disease Control and Prevention. 2014;18(05):411-414(in Chinese).

34. Xiang Q. Influencing factors of screening willingness for cervical cancer. Electronic Journal of Practical gynecology. 2019;6(20):10-11,20(in Chinese).

35. Wang X. Levels of Knowledge and Influencing Factors of Cervical Cancer Lesions in High-risk Women. Journal of Preventive Medicine of PLA. 2017;35(10):1264-1266,1297(in Chinese).

36. Du X, Zhang Y, Fu X, Li L, Meng Y. Levels of knowledge of and compliance with cervical cancer screening：a cross-sectional study of high·risk patients. Armed Police Medical. 2017;28(10):992-998(in Chinese).

37. Zhou M. Investigation on knowledge, attitude and practice of cervical cancer prevention and treatment and analysis of influencing factors. Nursing Practice and Research. 2019;16(13):96-98(in Chinese).

38. Zhu Y, Zhang J. Study on acceptance and influencing factors of cervical cancer screening adult women in Chengdu. Maternal and Child Health in China. 2014;29(26):4268-4271(in Chinese).

39. Song B, Di J, Ma L, Zhao Y, Wu J. Survey on status of awareness and practice of cervical cancer prevention and control among rural women in some areas of China. Health Education in China. 2018;34(12):1076-1080(in Chinese).

40. Zhao X, Wang Y, Liu Z, Duan X, Hu S, Wang Y, et al. Knowledge and its influencing factors of cervical cancer screening and human papillomavirus vaccines among 19201 Chinese population. Chinese Journal of Cancer Prevention and Treatment. 2022;29(09):623-629,649(in Chinese).

41. Zhang Z. Investigation on knowledge, attitude and practice of cervical cancer screening among 2000 rural women and its influencing factors. China Sanitary Engineering. 2021;20(03):416-418(in Chinese).

42. Wu X. Current status and influencing factors of cervical cancer screening in 1000 women. Jilin medical. 2018;39(05):891-892(in Chinese).

43. Zhou L, Lin W, Li J, Shi X, Yang M, Song Y. Current status and influencing factors of cervical cancer screening self-efficacy in 226 gynecological outpatients. Journal of nursing. 2018;25(18):64-67(in Chinese).
